# Deep reinforcement learning for self-tuning laser source of dissipative solitons

**DOI:** 10.1038/s41598-022-11274-w

**Published:** 2022-05-03

**Authors:** Evgeny Kuprikov, Alexey Kokhanovskiy, Kirill Serebrennikov, Sergey Turitsyn

**Affiliations:** 1 Novosibirsk State University, Pirogova str., 2, Novosibirsk, 630090 Russia; 2 Aston Institute of Photonic Technologies, Aston University, Birmingham, B4 7ET UK

**Keywords:** Mode-locked lasers, Solitons

## Abstract

Increasing complexity of modern laser systems, mostly originated from the nonlinear dynamics of radiation, makes control of their operation more and more challenging, calling for development of new approaches in laser engineering. Machine learning methods, providing proven tools for identification, control, and data analytics of various complex systems, have been recently applied to mode-locked fiber lasers with the special focus on three key areas: self-starting, system optimization and characterization. However, the development of the machine learning algorithms for a particular laser system, while being an interesting research problem, is a demanding task requiring arduous efforts and tuning a large number of hyper-parameters in the laboratory arrangements. It is not obvious that this learning can be smoothly transferred to systems that differ from the specific laser used for the algorithm development by design or by varying environmental parameters. Here we demonstrate that a deep reinforcement learning (DRL) approach, based on trials and errors and sequential decisions, can be successfully used for control of the generation of dissipative solitons in mode-locked fiber laser system. We have shown the capability of deep Q-learning algorithm to generalize knowledge about the laser system in order to find conditions for stable pulse generation. Region of stable generation was transformed by changing the pumping power of the laser cavity, while tunable spectral filter was used as a control tool. Deep Q-learning algorithm is suited to learn the trajectory of adjusting spectral filter parameters to stable pulsed regime relying on the state of output radiation. Our results confirm the potential of deep reinforcement learning algorithm to control a nonlinear laser system with a feed-back. We also demonstrate that fiber mode-locked laser systems generating data at high speed present a fruitful photonic test-beds for various machine learning concepts based on large datasets.

## Introduction

Laser systems are both important practical devices and complex physical systems where ML techniques can improve performance and offer control of the nonlinear dynamics of radiation. Designing ML algorithms for specific laser system requires rather elaborate efforts that includes data collection, signal processing, feature designing, tuning hyperparameters and so on. Most of the conventional ML approaches, both supervised and unsupervised learning, face various challenges when they are applied to building universal algorithms to control laser sources. The reason is that the process of improving laser performance is not straightforward and it requires to address sequential decision-making tasks involving set of trials. Thus, this technical and physical laser problem is perfectly suited for the application of the reinforcement learning technique, that has a potential to build the systems with the elements of the artificial general intelligence^1^. The fusion of the reinforcement learning and deep neural networks, called deep reinforcement learning (DRL) is a powerful alternative to the supervised learning, replacing learning from the labelled examples by the trial and error approaches^2,3^.

Reinforcement learning has recently been demonstrated to have a wide application in optics. RL algorithms was applied in laser beam welding processes^4^, for control optical tweezers^5^, for reconstructing an unknown quantum state^6^, and alignment of a seed laser for free-electron laser optimization^7^. In the scope of mode-locked lasers there are already promising application of DRL to control the output radiation. Most of them are related to lasers based on nonlinear polarization effect (NPE). Kutz et al^8^ demonstrated that deep Q-learning algorithm is capable to learn how to operate with bi-stability in fiber cavity in order to achieve stable mode-locking. The work^9^ demonstrates a possibility to stabilize mode-locked regime of NPE laser under temperature and vibratory disturbance by actor-critic DRL algorithm.

Here we apply the DRL technique to build self-tuning fiber laser source of dissipative solitons. Dissipative soliton (DS) is a particular example of the general concept of a “solitary wave”—that localized (in space or time), stable particle-like object can be formed by the nonlinear interactions of distributed waves (fields)^10–14^. Dissipative soliton occurs in the various nonlinear systems as a complex balance between both dissipative (e.g. amplification and loss) and conservative (e.g. dispersion/ diffraction and nonlinearity) effects. In the pulsed fiber lasers, for instance, initial growth of signal from noise is dominated by the balance between gain and losses. However, when laser signal power is stabilised at the certain level, effects of dispersion and Kerr nonlinearity together with the dissipative effects (filtering, gain/loss saturation and so on) shape of the form of the resulting pulse. Dissipative soliton is the important nonlinear science concept in the field of mode-locked lasers that has already made significant impact on the understanding of nonlinear interaction between light and matter and led to practical implementations^15–17^. Formation of DS involves dissipative processes, for instance, spectral filtration which stabilize the pulse from temporal and spectral stretching in a laser cavity. Similar to the soliton theory, the concept of DS is generic, and was demonstrated with different architectures of fiber-mode locked lasers^15,17^, micro-resonators^18,19^ and in other applications beyond optics^16^. Therefore, our results on the development of the controlling system that is capable automatically stabilize dissipative soltitons may potentially find applications in various fields. As a controlling tool for adjusting parameters of mode-locked pulses we chose spectral filtration. Recent interest in smooth spectral filtration inside fiber cavity has emerged, in particular, due to the possibility to generate complex temporal patterns, such as soliton molecules^20^.

In recent years the DRL has substantially advanced^21^. However, yet there are numerous challenges in applying the DRL algorithms to the real-world systems^22^, that, generally, such have continuous state and action spaces requiring vast amount of training procedures. In^5,23^ it was proposed to use the simulation environment to train the agent, allowing to apply different techniques for accelerating learning, for example, distributed learning^21,24^. Nevertheless, transfer learning using synthetic data has its own challenges and nuances. Here, to accelerate the learning process, we propose an approach that creates a simple model of the real system with deterministic dynamics, based on previously collected experimental data. This model is used to train the agent allowing to set an initial knowledge about the dynamics of the environment.

## Laser system

**Figure 1:**
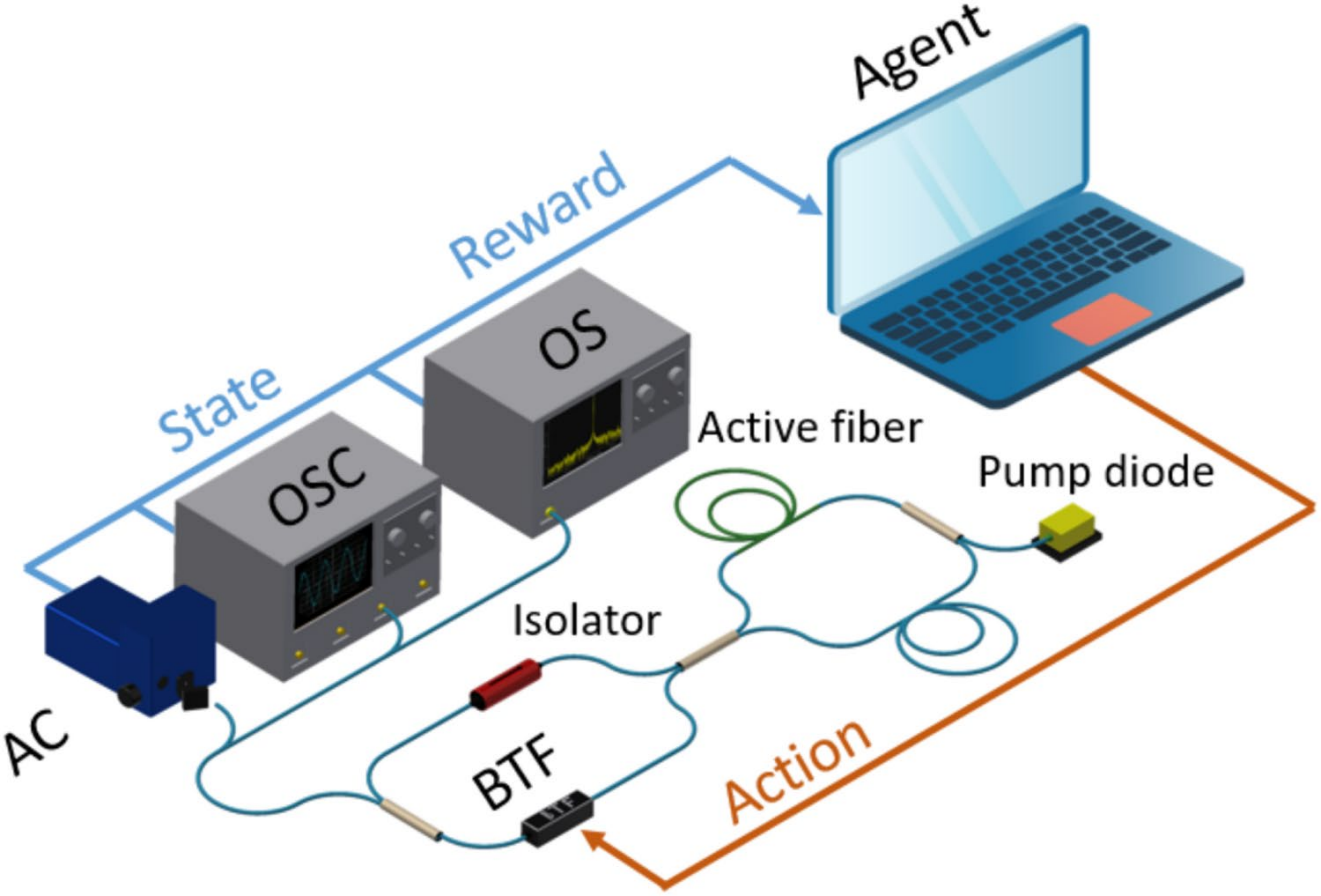
Experimental setup of the laser and measurement systems. *AC* autocorrelator, *OSC* oscilloscope trace, *OSA* optical spectrum analyser, *BTF* bandwidth tunable filter.

As an experimental source of the dissipative solitons to test the proposed technique we used figure-of-eight mode-locked fiber laser. This is a flexible platform to tune spectral-temporal properties of the dissipative solitons by adjustable amplification and a saturable absorption inside the fiber loops^25,26^. The fiber laser cavity consists of two fiber loops, unidirectional (main) and bidirectional (NALM) ones, connected to each other through a 40/60 coupler Fig. 1. NALM loop comprise 5-m long amplifying section of double-clad Yb-doped fiber with absorption of 3.9 dB m-1 at 987 nm. The main loop also includes a 40/60 output coupler and a high-power Faraday isolator that ensures unidirectional propagation. Active fiber is pumped through fiber beam combiner by multimode laser diode at 978 nm. All stretches of fiber and fiber elements inside the cavity maintain polarisation. We implemented a tunable spectral filter allowing to tune simultaneously the central wavelength and spectral bandwidth in the range of 1030 – 1070 nm and 2.4 – 4.2 nm, respectively.

The measurement system included the autocorrelator A.P.E pulseCheck for measuring the autocorrelation function (ACF), 16-GHz osciloscope Tektronix DPO71604C for measuring oscilloscope trace and the optical spectrum analyser Yokogawa AQ6370D with spectral resolution 0.02 nm for analysis of an optical spectrum.

To explore possible output regimes of the laser, we changed the bandwidth and the central wavelength of the spectral filter in the following order. First, we fixed pumping power of the laser cavity at 2 W. At a fixed spectral bandwidth 4.2 nm of the spectral filter, we gradually reduced the central wavelength of the filter from 1070 nm to 1030 nm with a step of 0.1 nm and measured the parameters of the output radiation at each step. Then the bandwidth of the spectral filter was reduced by 0.1 nm and the procedure was repeated until the spectral bandwidth became equal to 2.4 nm.

**Figure 2:**
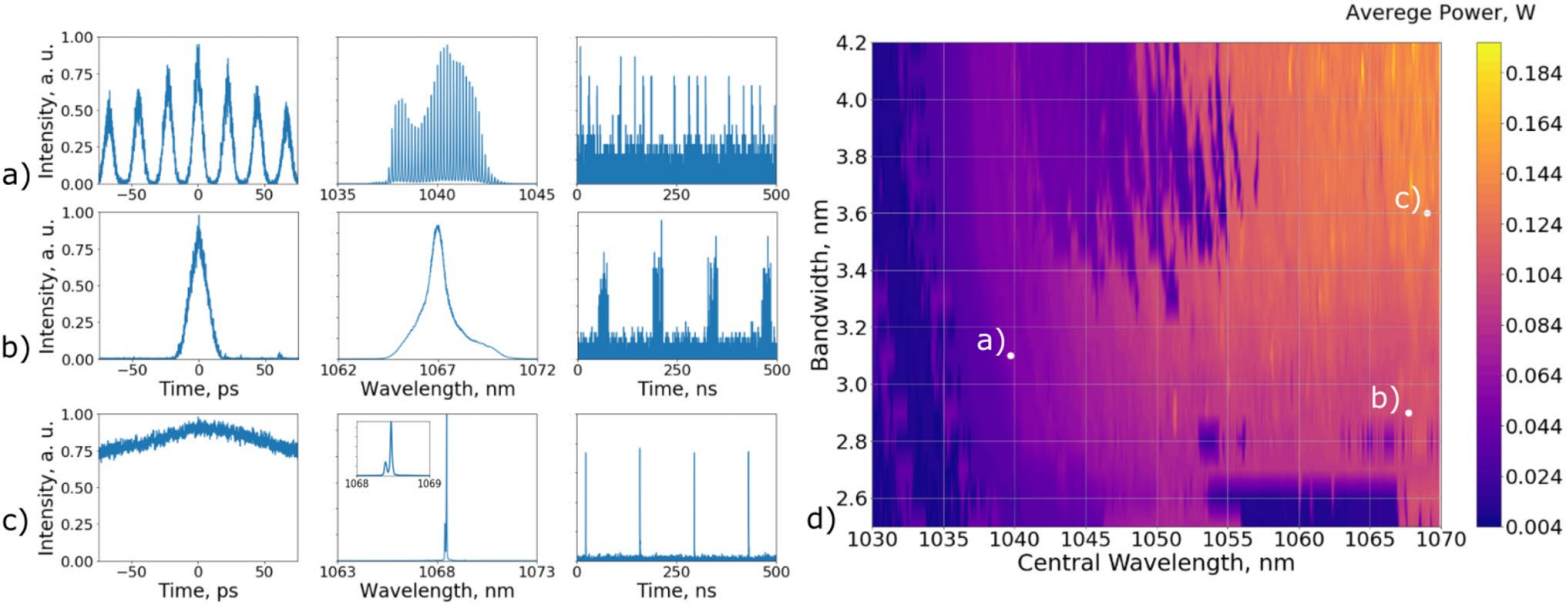
Examples of the three main pulsed regimes generated in the studied laser cavity. (**d**) Map of the average power of the output radiation; Autocorrelation function, optical spectrum and oscilloscope trace of (**a**) soliton molecule (**b**) partly coherent multi-pulses (**c**) single pulses.

Figure 2 illustrates the characteristic regimes from the laser operation. We distinguish three key types of the output signals: (a) soliton molecule (b) multi-pulsing regime (c) single pulse DS. The energy of pulses ranged between 61.5 and 455.5 mW . The spectrum width of the output regimes were 0.21 - 4.7 nm. ACF duration of single pulse DS varied from 14 to 513 ps.

In the current work, the goal for RL algorithm was to reach stable single pulsed regime.

## Deep Q-learning algorithm

We formulated the problem in terms of RL by describing the laser tuning process as a sequence of changes of the filter’s width and central wavelength by certain values. The central length varied between 1030 nm and 1070 nm, the width - from 2.5 nm to 4.2 nm, the variation step was 0.1 nm for both parameters. Thus, the agent’s action space consisted of four possible actions: ±0.1nm for width and ±0.1nm for central length. When the agent attempted to go outside the range of the acceptable values, then the action was not performed and the agent remained in the same position. The state space of the laser was described using two spectral filter parameters and five output radiation characteristics: power and width of the spectrum, the noise and duration at half maximum of the autocorrelation function, the amplitude of pulses.

In the reinforcement learning, training of an agent consists in searching for an optimal policy by evaluating the results of the agent’s interaction with the environment. We used the DDQN algorithm^27,28^ (which we describe in detail further in the article) in which the policy is determined by computing the value of the action state function (Q-function). At each step, the agent estimates the value of the Q-function for each possible action and chooses the action with the maximal value. As the state space in our problem was continuous, we used a deep neural network to approximate the value of the Q-function. The training process consisted of direct interaction of the agent with the environment and recalculation of the values of the Q-function until the agent learned the optimal policy.

Commonly, there is an interest in finding stable pulsed regime with the highest energy provided by the fiber laser. However, energy of a pulse has a threshold value, above which mode-locked regime switches to unstable or partially mode-locked regime^29^. Therefore, we apply here the following reward:

1 $$\begin{aligned} R = \frac{P_{average}}{P_{noise}} \end{aligned}$$

where $P_{average}$ is the pulse energy taken from the oscilloscope trace, $P_{noise}$ is the characteristic of the pulse noise, which is obtained from the ACF data. $P_{noise}$ was calculated as a normalized difference between a height of ACF trace and a height of envelope of ACF trace. To derive the height of ACF envelope, we applied a low-pass 3-order Butterworth filter with 0.01 ($\pi$ rad/sample) cut-off frequency to ACF trace.

Similar approach was used for the choice of the objective function for optimization problem^30^ and reward for reinforcement learning task^8^. However, in these papers it was proposed to use the fourth-moment of the Fourier spectrum of the waveform. This works nicely in the numerical modeling, however, it is challenging to measure the waveform experimentally. Therefore, here as an alternative possibility, we propose to use $P_{noise}$ instead: as well as the fourth-moment, it has high value for chaotic solutions, and is smaller for the desired mode-locked states, but it is easier to measure.

The training was divided into sessions. Each session started with randomly selected parameters of the spectral filter and continued until the agent performed 200 actions. The purpose of the algorithm was to find the optimal policy, under which the agent collected on average the highest total reward for the session.

Since training an agent on a real system takes a lot of time, we proposed to use a simplified model of the considered system as a training environment. This model presented itself a two dimensional map with axes corresponding to the spectral filter parameters (width and central wavelength). For each pair of these parameters, we measured five characteristics of the state in the real system: power and width of the spectrum, the noise and duration at half maximum of the autocorrelation function, the amplitude of pulses. Measurements have been performed at fixed currents of pumping diodes for the admissible values of the spectral filter parameters. In our model, the agent was able to move in four directions, at each step receiving the characteristics of his position form our regime map.

This model had several noteworthy differences with the real system. First, the map that was used to build the model was collected as described in the first chapter, so for each state we had only one true transition to another. However, the proposed model allowed one to choose any of the four actions during the interaction of the agent with the model. Second, the laser itself is a complex nonlinear dynamical system in which, in addition to noise, next states depended on the previous ones. The model, on the other hand, was a deterministic system, in which not only the number of states was finite, but all transitions between states were predetermined. However, still learning in such a simplified environment does make sense. In addition to dramatically reducing training time, it allowed the agent to study the transitional states from unstable to the mode-locking regimes since the used data was obtained from a real system. The agent also can continue training on a real system to learning about more complex practical dynamics.

Training and evaluation of the reinforcement algorithm were running on computer system consists of intel core i5 8800 (2.8 GHz), 32 Gb of RAM and Nvidia 1060 6 Gb video card. The training time was up to 3 hours. The model of a real system consisted of 6800 unique regimes. The laser adjustment time was up to 2 minutes.

## Results and discussion

First, we demonstrated the process of training of a deep reinforcement learning agent on a model of environment. Next, we used a trained agent to tune a real laser system. Finally, we modified the environment by changing the pump current of the laser diode and showed that the strategies that the agent has learned also allow tuning of such systems.

**Figure 3:**
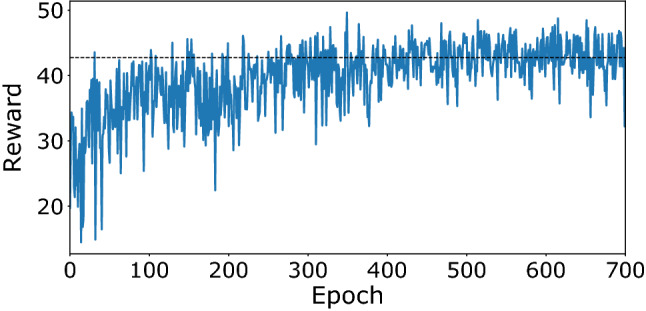
The variation of the rewards during training on the model of environment.

We collected the data of the regimes for the range of admissible values of the spectral filter at a fixed value of the pump current of the laser diode of 2.7 A. The model of the environment was created using the measured data. Figure 3 shows variations of reward during the training process of the deep RL agent. In this case, one epoch consisted of 100 sessions with 200 actions each. The session started with a random initial value of the spectral filter parameters, which made the learning curve look noisy. The graph shows that after the 400th epoch, the algorithm gained on average a cumulative reward equal to 42. It should be noted that since the agent received a reward at each step, the learning curve starts from a nonzero value.

**Figure 4:**
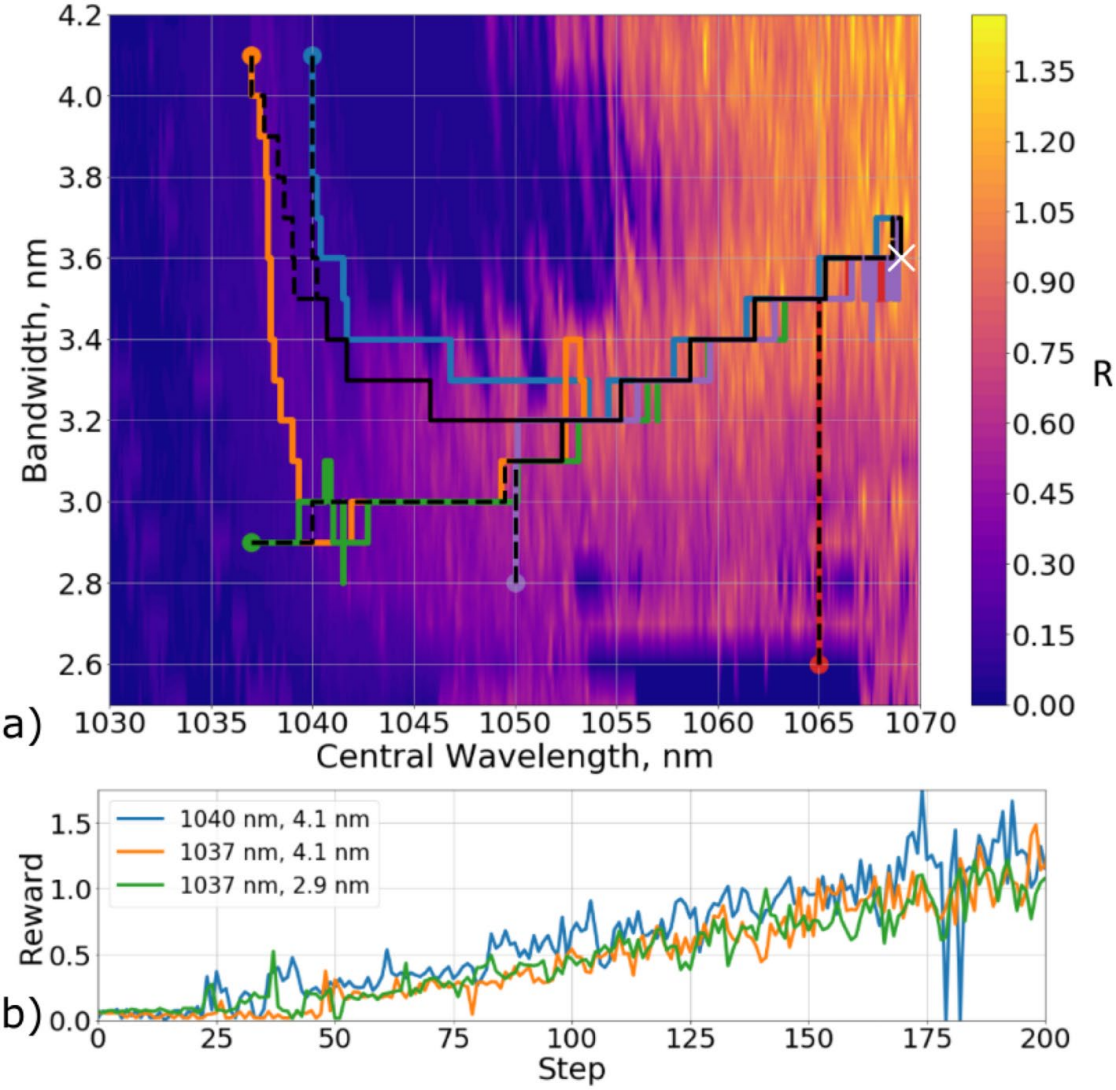
(**a**) Trajectories of the agent’s movement, represented on a two-dimensional map of spaces of admissible filter values. The color of the map shows the values of the instant reward, which was calculated on the collected data at a current of 2.7 A. The black dotted lines show the agent trajectories obtained on the model of the environment. The colored lines show the trajectories obtained on the real laser system. (**b**) The value of the immediate reward depending on the step number of the agent for three trajectories.

Because the laser was controlled by varying only two parameters, the data itself, as well as the trajectory of the laser adjustment, can be displayed on a two-dimensional map. In Fig. 4a, the color indicates the value of the reward function *R* for collected data at the pump current 2.7 A. The black dotted lines show the agent trajectories obtained on the model of environment. Since the model was a deterministic system, these trajectories converge into one, and the trajectories for the same starting points will always repeat themselves.

In the Fig. 4a, colored lines represent the trajectories of the agent when tuning a real laser system. The starting points were chosen in such a way that they had non-pulsed generation regimes. One can notice that even though the starting positions of the from the model and from the real system were the same, the trajectories themselves were different. This is because the real environment is a stochastic system with unknown dynamics of transitions from one state to another further complicated by the presence of noise. Despite this fact, the deep reinforcement learning agent was able to tune a real laser system. Figure 4b shows the dependency of the immediate reward on the step for three trajectories from the real system. One may notice that the reward increases throughout the tuning session.

**Figure 5:**
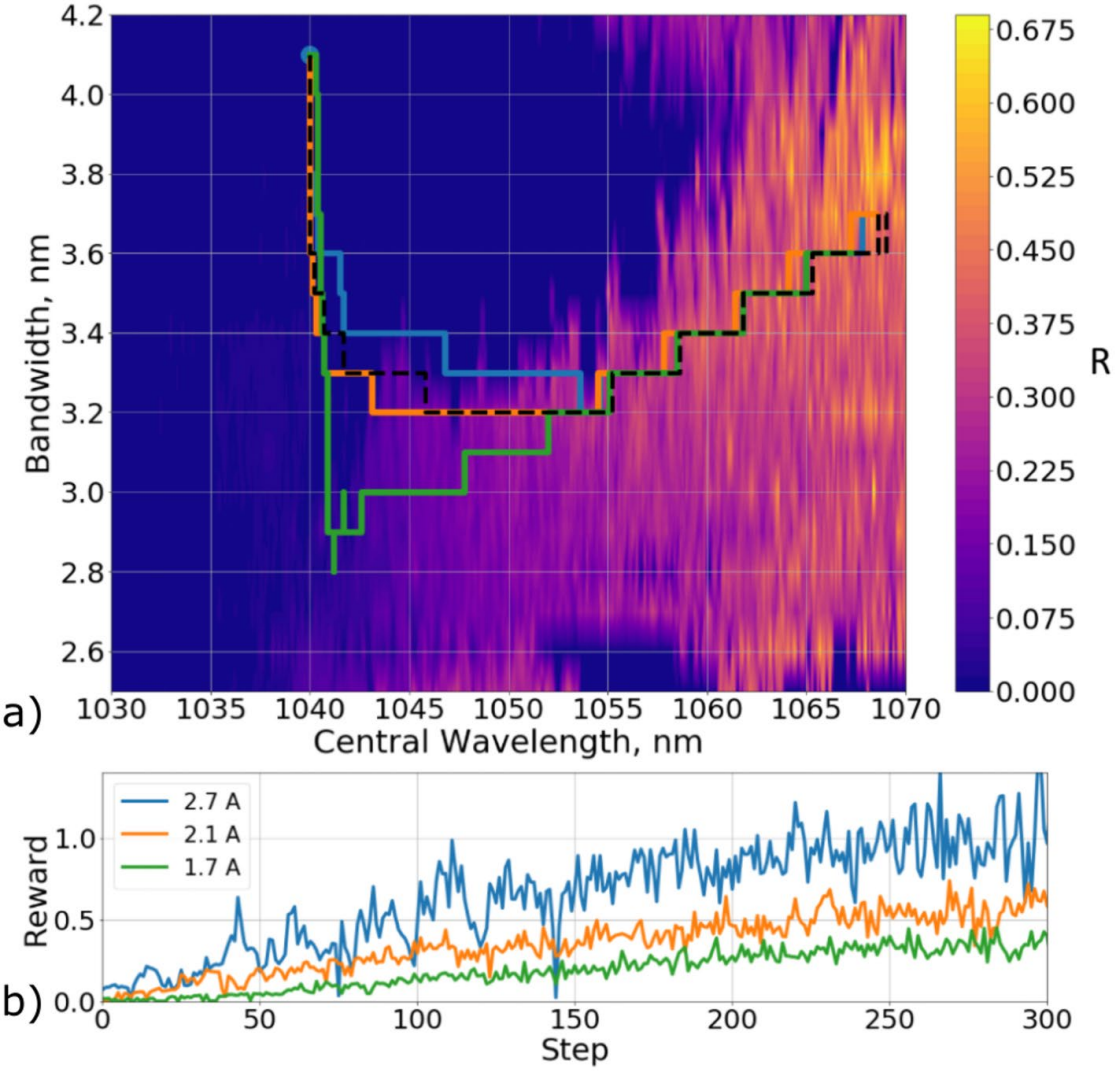
(**a**) The trajectories of the agent, presented on a two-dimensional map of the spaces of admissible filter values, which were obtained at different values of the pump current. Blue line - agent trajectory for 2.7 A environment, orange line - agent trajectory for 2.1 A environment, green line - agent trajectory for 1.7 A environment, doted line - agent trajectory for the model of environment. The color of the map shows the values of the immediate reward, which was calculated on the collected data at a pump diode current of 2.1 A. (**b**) The value of the immediate reward depending on the step number of the agent.

After computing the weights of the deep RL agent’s neural network at the laser diode pumping current of 2.7 A, we applied this agent to environments with different currents - first to 2.1 A and then to 1.7 A. We demonstrated that even in these cases the agent was able to find mode-locking regimes. Figure 5a shows the trajectories that were obtained for different pump currents of the laser diode with the same starting point of the system. To demonstrate the difference of the environment for the pump current different than 2.7 A, Fig. 5a shows a map corresponding to the current of 2.1 A. It should be noted that since the reward depends on the pulse power (Eq. 1), an increase in the pump gain leads to a decrease of the reward.

Comparison of the trajectories of the agent in Fig. 5a shows that starting from about the 150th step, they are close. At this stage, the agent has already found a stable mode locked regime, and continued to search for a state with a maximum reward. However, in the beginning the trajectories are very different despite the same starting point. The reason is that the algorithm tries to cling to a stable generation regime, but the stable lasing field decreases with the decay of the pump current, which can be seen through the comparison of Figs. 4a and 5a. The trajectories in Fig. 5a show that the algorithm adapts to these changes and allows tuning the laser even in the case of the changed environment.

### Discussion

Deep Reinforcement Learning is a powerful tool that can be used to setting up a laser system. In this paper, we have demonstrated how DDQN algorithm may be implemented for self-tuning task of an experimental mode-locked fiber laser with a nonlinear loop mirror. The algorithm successively have found physical patterns. In our laser, the spectral profile of gain and losses is unevenly distributed along the wavelength and reaches its maximum at a wavelength of 1068 nm. Based on the results obtained, we conclude that the algorithm has mastered this feature of the system and, when tuning, tried to find solutions in the vicinity of this wavelength. Note that Figs. 4 and 5 show that the steepest path from the initial point to the resulting solution is a straight line. However, this trajectory passes through a large area of unstable generation. Too large or too narrow band-passes of the filter lead to unstable pulse generation or generation of the soliton molecules. Considering the tuning trajectories, the algorithm learned to bypass these areas, which allowed it to find the area of stable solutions immediately and continue tuning already in it. In this work, we used the universal reward $P_{average}/P_{noise}$, which is not tied to a certain laser. Thus, the proposed deep reinforcement learning algorithm can potentially be used to solve the problem of self-tuning of other laser systems. We anticipate that further study will unlock true potential of the proposed technique in the complex laser systems.

## Methods

### Reinforcement learning

Reinforcement Learning (RL) is a field of machine learning in which an agent is trained in an environment so that its behavior maximizes the cumulative reward. RL can be attributed to one of the machine learning paradigms, along with supervised learning and unsupervised learning^2^. In addition to concepts such as agent and environment, reinforcement learning theory uses terms such as policy $\pi$ and reward signal *R*. The policy defines the way a learning agent behaves at a given moment. In other words, politics is a mapping of perceived states of the environment and the actions to be taken in those states. The reward signal defines the goal of the reinforcement learning task. At each time step, the environment sends a single number to the agent, called a reward. The agent’s sole goal is to maximize the overall reward he receives over the long term. Thus, the reward signal determines which events are good and bad for the agent.

### Q-learning

Q-learning is an off-policy temporal difference (TD) learning algorithm that approximates the value of a state-action value function or Q-function based on previously obtained estimates of this function. The Q-function is defined as the expected discounted reward that an agent will receive when starting the game in states *s* with action *a* and then acting following policy $\pi$. Mathematically, it can be described in the following way:

2 $$\begin{aligned} Q^{\pi }(s, a) = {\mathbb {E}}_{\pi }[R_t|s_t=s, a_t=a] \end{aligned}$$

where $R_t$ is the expected reward that we will receive at the end of the session. The Q-function determines the performance of an agent performing a certain action and moving from the current state to the next one with the policy we have chosen. The policy itself is defined as *argmax*(*Q*(*s*, *a*)) for all possible actions. During training, the agent learns and converges on the optimal policy that maximizes the total reward that can be obtained during one game episode. The Q-function itself can be written out recursively:

3 $$\begin{aligned} Q(s, a) = r(s, a) + \gamma \max _{a'}Q(s', a') \end{aligned}$$

where *r*(*s*, *a*) is the reward we will receive if we move from state *s* to state $s'$ by acting *a*, $\gamma \in [0,1]$ is a discount factor that determines the relative importance of future and immediate rewards. In order for the agent to take different actions during the learning process, thereby exploring the environment, an $\epsilon$-greedy strategy is used to select an action. During the episode, the agent chooses actions with the highest value of the Q-function in state *s* with probability $(1-\epsilon )$ or randomly with probability $\epsilon$, where $\epsilon \in [0,1]$. After the agent, as a result of action *a*, has passed from state *s* to state $s'$, and has received a reward *r*, the value of the Q-function is updated using the following formula:

4 $$\begin{aligned} Q^{new}(s, a) = Q(s, a) + \alpha (r + \gamma \max _{a'} Q(s', a') - Q(s, a)) \end{aligned}$$

where $\alpha \in [0,1]$ is the learning rate. In discrete environments, with a finite number of states and actions, the Q-function is represented in the form of a table, and the learning algorithm itself consists of recalculating this table by the formula until we get the optimal value of the Q-function.

### Deep Q-learning

We obtain a deep reinforcement learning algorithm when we use deep neural networks to approximate the policy, Q-functions or anther RL function^3^. This approach is used when we are dealing with continuous environments in which the number of states or actions is unlimited. In the Deep Q-Network (DQN) algorithm, neural networks (NN) are used to approximate the values of the function $Q (s, a, \theta )$, where the parameters $\theta$ are the weights in the deep neural network^31^. In our case, we use a multilayer neural network, which receives states *s* as input, and the output of this NN is a vector of values $Q(s, a_i, \theta )$ for each of the possible actions $a_i$.

**Figure 6:**
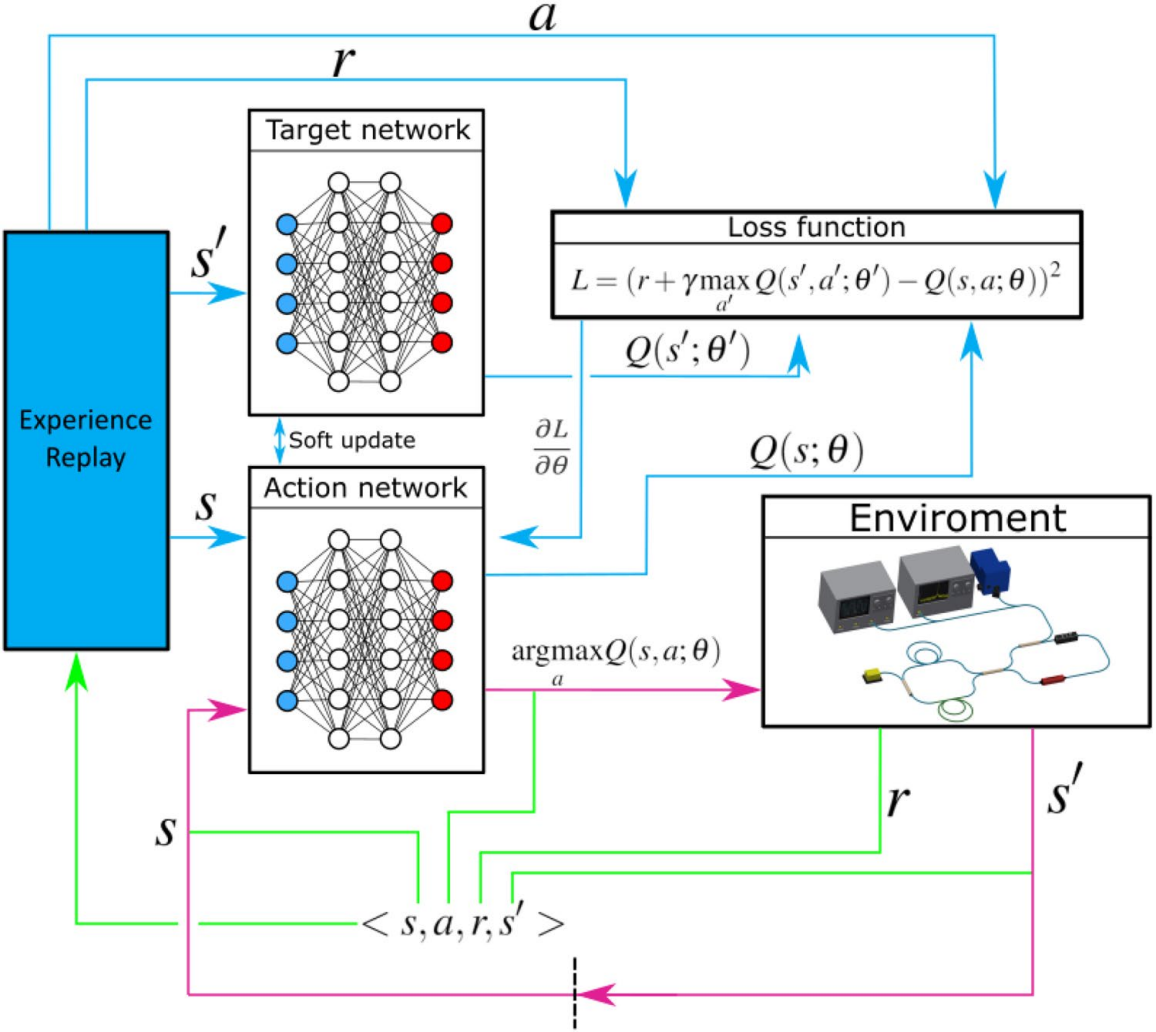
The architecture of double deep Q-learning network with experience replay buffer. Purple arrows show the agent’s interaction with the environment. The dotted line separates the time steps. Green lines show what is saved in the experience replay buffer and at what stages. Blue arrows describe the agent training process.

The neural network is trained using the backpropagation method, where the loss function is described as the square of the difference between *Q*(*s*, *a*) and $Q^{new}(s, a)$, which is obtained from equation (4):

5 $$\begin{aligned} L = (r + \gamma \max _{a'}Q(s', a'; \theta ') - Q(s, a; \theta ))^2 \end{aligned}$$

where $\theta$ and $\theta '$ are the weights of two neural networks of the same architecture which are called action network and target network respectively. The action network is updated during error propagation training, and the target network is updated by copying the weights $\theta$ of the action network every few episodes. This approach is called Double Deep Q-learning network (DDQN) and allows to avoid overestimating the action-state function in the learning process^28^. Also, the experience replay buffer are used to destroy the correlation in data and make it possible to use old experiences in Off-Policy algorithms^32^. The DDQN architecture with experience replay buffer is shown in Fig. 6.

This scheme consist of three parts. The process of the agent’s interaction with the environment is depicted by purple arrows. The state of the environment is given to the input of the action neural network, which predicts the values of the Q-function for all possible actions, after which the action with maximum value of the Q-function is selected. The green arrows show the process of saving experience into the experience replay buffer, which is then used to train the agent. The agent training process is represented by blue arrows. The data from the experiment replay buffer is used to calculate the loss function and further the gradient step.

## Data Availability

All datasets used and/or analysed during the current study available from the corresponding author on reasonable request.
